# Symptom management, adherence to therapy, and filling the gaps of medical cannabis therapy: a qualitative study on the importance of nursing consultations for fibromyalgia patients

**DOI:** 10.1186/s42238-025-00346-z

**Published:** 2025-10-29

**Authors:** Giulia Martina Bassi, Valeria Giorgi, Michela Lazzarin, Ramona Meanti, Robert J. Omeljaniuk, Piercarlo Sarzi-Puttini, Antonio Torsello

**Affiliations:** 1 School of Medicine and Surgery, University of Milano-Bicocca, Monza, Italy; 2 Moncucco Hospital Group, Lugano, Switzerland; 3 University of Applied Sciences and Arts of Southern Switzerland – SUPSI, Manno, Switzerland; 4 Department of Biology, Lakehead University, 955 Oliver Rd, Thunder Bay, ON P7B 5E1 Canada; 5 Rheumatology Department, IRCCS Ospedale Galeazzi-Sant’Ambrogio, Milan, Italy; 6 Department of Biomedical and Clinical Sciences, University of Milan, Milan, Italy

**Keywords:** Fibromyalgia, Medical cannabis, Integrative medicine, Education, Nursing, Qualitative study

## Abstract

**Background:**

In agreement with the latest European League Against Rheumatism (EULAR) on patient education for fibromyalgia (FM) treatment, nurses should be involved in the therapeutic plan of FM patients to provide information about the disease and pharmacological and non-pharmacological approaches that can be used to mitigate symptoms, including the use of medical cannabis.

This study aims to demonstrate the key role of nursing educational intervention to improve self-care and therapy adherence by FM patients.

**Design, setting, participants and interventions:**

All potential subjects, solicited from the Italian Fibromyalgia Syndrome Association (AISF Onlus) address book (*n* = 1100), were provided with a description of the study as well as a privacy protection form (as an e-mail) subsequent to authorization from the AISF. In this qualitative study, nurse educational intervention (30 min duration) was offered via videoconference to FM patients who were taking and/or had taken medical cannabis as well as patients not in therapy but interested in taking it, who matched the inclusion criteria. Two weeks before the educational intervention, subjects (*n* = 30) completed the Revised Fibromyalgia Impact Questionnaire (FIQR) and the A-14 Scale on-line. Two weeks following intervention, subjects repeated the FIQR and A-14 Scale in addition to the Clinical Global Impression - Global Improvement (CGI-I) Scale.

**Results:**

Historic diagnoses of subjects included terms such as “insane”, “imaginary ill”, and “whiny”; as well, their physical conditions were underestimated by their immediate families. All subjects have problematic employment histories which consistently identified their varied employments as physically too demanding. Over time, increased physician- and societal-awareness, resulted in all subjects being diagnosed with FM; consequently, all subjects reported a strong desire to become well-informed about FM and its treatment. Although medical cannabis (MC) was an available therapy, twenty subjects reported that cannabis had never been proposed despite years of ineffective therapies.

**Conclusions:**

The subjects were disappointed and discouraged by the widespread ignorance about their condition and the lack of recognition by the National Health System. Our findings suggest that patients with FM require specialist clinical advice from initial diagnosis through to the end of treatment, and that nurses with in-depth knowledge of fibromyalgia and treatment options are the best professionals to perform this task; furthermore, healthcare professionals should receive a better education about MC-based treatment regimens.

**Registration:**

This study protocol conforms to the Declaration of Helsinki and was approved by the Ethics Committee of the University of Milano-Bicocca (Protocol 545 9-7-2020) and registered on Clinicaltrials.gov NCT05247411. Written informed consent for participation in the study and publication of the results was obtained from all respondents.

## Introduction

Fibromyalgia (FM), or fibromyalgia syndrome, is a chronic condition characterized by a spectrum of symptoms that include: (i) chronic generalized pain, (ii) fatigue, (iii) sleep disturbances and (iv) high psychological distress, up to mood disorders, causing impaired functionality and quality of life (Clauw 2014). The global prevalence of FM is estimated to approximate 2% (Queiroz 2013), mainly affecting women between the ages of 30 and 55 (Sumpton & Moulin 2014). Its pathogenesis is still largely unknown; however, a crucial role has been recognized both in “top-down” (cognitive-emotional, linked to resilience), and in “bottom-up” mechanisms (inflammatory-painful) (Sarzi-Puttini et al. 2020), which lead to a decrease of the nociceptive threshold at a central level thereby creating the newly termed “nociplastic pain” (Trouvin & Perrot 2019).

### Conventional treatments

Currently, the European League Against Rheumatism (EULAR) offers no definitive treatment of fibromyalgia; by comparison, Macfarlane et al. (2017) suggest a gradual and multimodal approach. This approach includes patient education about the disease and its management and, if this is insufficient, its integration with (i) non-pharmacological treatments (primarily, physical activity) and (ii) pharmacological treatments.

The pharmacological treatments currently available include antidepressants, muscle relaxants, antiepileptics and, to a lesser extent, analgesics (Sarzi-Puttini et al. 2020); recently, however, cannabinoids have also been considered a possible treatment for patients with inadequately controlled symptoms (Yassin et al. 2019; Chaves et al. 2020; Giorgi et al. 2020). In general, as the most appropriate therapeutic approach should be multidisciplinary, based on an individualized program of care that includes the general practitioner, the inclusion of a specialist and a nurse is widely accepted (Stournaras and Petrovic 2019). Various factors can exacerbate symptoms, including the presence of co-morbidities that further limit quality of life and functionality (Lichtenstein et al. 2018). Notably, inadequate patient education and awareness as well as flawed compliance with pharmacological therapies may result in poor treatment outcomes (Ben-Ami Shor et al. 2017). In particular, it has been shown that increasing the patient’s knowledge of the disease improves the management of pain-related symptoms (Conversano et al. 2019); to illustrate, several studies have suggested that a specific educational program performed by a nurse to help the patient in the management of his condition and therapy can significantly improve the outcome (Hughes et al. 2016; Balneaves et al. 2018; Conversano et al. 2019). Therefore, the nurse can help FM patients not only to increase self-care and awareness, to cope with a chronic disease, but also to fill the knowledge gaps and barriers to therapy with, for example, medical cannabis (MC) (Balneaves et al. 2018).

### Medical cannabis in FM

In Italy, the regulation of medical cannabis use is defined by the Decreto Ministeriale 09/11/2015 according to which its use is limited to the treatment of “chronic pain and pain associated with: (i) multiple sclerosis as well as spinal cord injury, (ii) nausea and vomiting caused by chemotherapy, (iii) radiation therapy, (iv) HIV therapies. Medical cannabis may also be used as an appetite stimulant in (i) cachexia, (ii) anorexia, (iii) loss of appetite in patients with oncology or AIDS and (iv) anorexia nervosa. In addition, medical cannabis may be used to offset the hypotensive effect in glaucoma as well as the reduction of involuntary body and facial movements associated with Gilles de la Tourette syndrome”.

The NHS also approves the use of MC for treatment of chronic pain unresponsive to nonsteroidal anti-inflammatory drugs, steroids, or opioids.

The few studies which have described the use of cannabis in patients with FM, are symptomatic of a general lack of information about the type, dosage and long-term adverse effects of MC (Fiz et al. 2011; Piper et al. 2017; van de Donk et al. 2019; Strand et al. 2023).

Recently, one retrospective study examined the analgesic efficacy of MC and adverse events in Italian adult patients diagnosed with FM and who were resistant to conventional drugs. The data obtained from medical reports archived in the pain clinic of Nuovo Ospedale degli Infermi of Ponderano (Biella, Italy), assessed between June 1, 2016, and October 31, 2018, demonstrated that MC therapy significantly reduced pain intensity proportional to the duration of treatment (1, 3 and 12 months) and were accompanied by few serious adverse effects (Mazza 2021).

Although MC seems to offer a clinical advantage in terms of efficacy and pain reduction, further studies are required to establish the best therapeutic strategy in terms of posology, the phytocannabinoids content, and treatment duration (Giorgi et al. 2020).

## Methods

### Aims

This study aims to elucidate the essential role of nursing intervention in order to improve symptom control and compliance in fibromyalgia patients to pharmacological and non pharmacological existing strategies, with a specific focus on those FM patients who use therapeutic cannabis, or are interested in taking it, by introducing a consulting nurse in the therapeutic path of patients.

After increasing patient knowledge of FM (in terms of symptoms, onset, treatment strategies that can improve the quality of life), self-care and adherence through the interview, the efficacy of the nursing educational intervention were evaluated using validated clinimetric scales.

### Design

We performed a qualitative study with face-to-face semi-structured interviews and patient-tailored educational interventions. Two weeks before the interview, subjects were asked to complete the Revised Fibromyalgia Impact Questionnaire (FIQR) and the A-14 Scale online. Two weeks after the interview, patients were asked to complete the same questionnaires in addition to the Clinical Global Impression - Global Improvement (CGI-I) Scale. This study adheres to the Consolidated criteria for Reporting Qualitative research (COREQ) (Tong et al. 2007).

### Enrollment strategy

The invitation to participate in the study was made by sending an e-mail to 1100 subjects among those in the database of the Italian Association of Fibromyalgia Syndrome (AISF - Voluntary Organization). To safeguard the privacy of patients, we used a database that included the e-mail addresses of physicians, nurses, friends of the AISF, and fibromyalgia patients. AISF provided only the e-mail addresses but no other data about name, sex, medical conditions, profession. The invitation briefly presented the study; attachments included a thorough explanation of the project, as well as an informed consent form for the processing of personal data and for the protection of privacy in case the patient wanted to participate in the study (with reference to EU Regulation 679/2016, Legislative Decree 196/2003 and subsequent amendments regarding the processing of personal data). Subjects who volunteered to participate had to reply to the invitation electronically and express their interest to be contacted.

Inclusion criteria chosen for enrollment included: (i) diagnosis of fibromyalgia syndrome carried out for more than 3 months and confirmed by the specialist (rheumatologist/algologist), (ii) age > 18 years, and (iii) ability to understand the objectives and methods of the study and, to read and sign the informed consent form.

To guarantee anonymity, each enlisted person was assigned a unique numerical code that was not associated with personal data, so that it was impossible to associate the answers provided in the questionnaires with a specific patient, but only to permit association of the first with the second questionnaire (pre/post).

### Data collection

After obtaining written informed consent for participation in the study, each participant was asked to complete the following online questionnaires:

I. collection of personal, biometric and clinical data (Age, Gender, Education level, Marital status, ongoing pharmacological and non-pharmacological treatments);
II. Revised Fibromyalgia Impact Questionnaire (FIQR) (Salaffi et al. 2013): disease-specific questionnaire which is composed of 21 elements that refer to the previous 7 days. The result of the FIQR is calculated as the sum of the scores of the three domains (physical function, general health and symptoms) in the following way: first, the score for the physical function domain is divided by 3, next, the score of the general health domain is left unchanged, and finally, the score of the symptom domain is divided by 2;
III. The A-14 Scale (Jank et al. 2009): is a specific questionnaire for the description of therapeutic adherence and individual barriers. It is made up of 14 questions, which can be answered using a 5-element Likert-scale (from “never” (5) to “very often” (0). The result of the scale ranges from “non-compliant” (score < 50) to “compliant” (score 50–56).

Two weeks after completing these online questionnaires, a Skype-videoconference, which included a 30 min educational intervention, was held with each subject. Two weeks after the videoconference, patients were required to complete the FIQR, The A-14 Scale, as well as the Clinical Global Impression - Global Improvement (CGI-I) Scale (which measures the subjective progress of the patient) (Busner and Targum 2007), to assess effects of the educational intervention on therapeutic perspective and compliance.

### Characteristics of the educational intervention

During the videoconference, the following items were investigated and evaluated:

I. Brief assessment of knowledge of FM: knowledge of symptoms, onset, treatment, strategies and non-pharmacological interventions that can be implemented to improve the quality of life (physical exercise, nutrition, participation in meditative movement practices, mutual aid groups, membership in a patient association, etc.);
II. Any MC intake or interest in starting the intake of MC;
III. Knowledge of MC therapy: specific knowledge of the drug, correct intake, adherence, treatment of side effects related to intake and strategies to reduce them;
IV. Self-care: how the person manages his personal life despite a chronic disease, how she/he monitors, recognizes and treats the symptoms in terms of timing and quality; possible presence of caregivers in their life who help in the management of the disease; employment and how FM affects it and daily life.

Skype teleconferencing was used for the educational intervention in order to reach a larger number of participants and in recognition of societal restrictions associated with the post COVID-19 emergency. Therefore, the educational intervention was structured as follows:

- Introduction to FM and CM to verify the person’s knowledge;
- Education about the correct management of therapy and side effects;
- Concluding part to resolve doubts and/or questions. 

### Ethical considerations

This study protocol conforms to the Declaration of Helsinki and was approved by the Ethics Committee of the University of Milano-Bicocca (Protocol 545 9-7−2020) and registered on Clinicaltrials.gov NCT05247411. Written informed consent for participation in the study and publication of the results was obtained from all respondents.

### Data analysis

Descriptive analyses were performed on study participants. Categorical variables were expressed as numbers and percentages, and comparisons of data obtained at the start and end of the study were made using the Student’s T-test. The correlation analysis between the analyzed variables was performed by calculating the Pearson correlation index. Values of *P* < 0.05 were considered statistically significant.

## Results

### Biometric and personal data

Of the 53 volunteers (1100 invitees), 23 were excluded as they were not in MC therapy and/or not interested in starting the therapy; consequently, 30 interviews were scheduled. Of these subjects, 8 were taking MC-based medications, and 2 had previously taken them but discontinued therapy. 20 patients had no prior experience with MC-based preparations but expressed interest in this form of therapy and sought additional information.

Subject characteristics are summarized in Table 1.

 Of the 30 subjects, 25 were receiving multidrug therapy including: (i) antidepressants (*n* = 6), (ii) analgesics (*n* = 18), (iii) NSAIDs (*n* = 11), and (iv) opioid analgesics (*n* = 8); by comparison, a small minority (*n* = 5) had abandoned all types of drug treatments due to adverse events.

**Table 1:** Participants’ characteristics

**Characteristics**	**N (%)**
Gender	
Women	24 (80)
Men	6 (20)
Age in years	
18–30	1 (3.3)
31–40	0 (0)
41–50	6 (20)
51–60	20 (66.6)
> 61	3 (10)
Martital status	
Single/Single	4 (13.3)
Married	20 (66.6)
Engaged	0 (0)
Cohabitant	2 (6.6)
Widower	1 (3.3)
Divorced	1 (3.3)
Free status following dissolution of union	2 (6.6)
Free status following death	0 (0)
Education	
Primary school	0 (0)
Secondary school	3 (10)
High school	15 (50)
Diplomas from institutes of higher artistic, musical and choreutic training	4 (13.3)
Academic Degree	7 (23.3)
PhD	1 (3.3)
Infancy educator	1 (3.3)

### Pre-intervention clinimetric data

Results of the pre-interview Revised Fibromyalgia Impact Questionnaire Italian (FIQR) and their relative severity index (Salaffi et al. 2013) are listed in Table 2.

**Table 2:** Mean, standard deviation and numerosity of pre-intervention FIQR and relative severity index

Severity Index	Patient code	FIQR Result pre-intervention	Numerosity
Remission	15	10.5 ± 0	1
Mild	4–5–8–19−22	42.9 ± 3.3	5
Moderate	1–2–3–13–20–24	57 ± 6.4	6
Severe	9–10–12–14–16–17–21–23–26–27–29–30	73.7 ± 4.2	12
Very Severe	6–7–11–18–25–28	87.3 ± 2.6	6

Subjects identified their severity index as: (i) “very severe” (*n* = 6), (ii) “severe” (*n* = 13), (iii) “moderate” (*n* = 6) and, (iv) “mild” (*n* = 5). The average score in the FIQR is 65.81.

Therapy compliance was resolved as (i) “Adherent” with an A-14 score of 50 to 56 (*n* = 8), and (ii) “Non-adherent” (*n* = 22) (Table 3).

**Table 3:** Mean, standard deviation and numerosity of A-14 scale questionnaire pre-educational interview

Adherence	Patient code	A-14 Scale Result	Numerosity
Adherent	4–5–6–11–14–15–24–26	52.8 ± 2.1	8
Non adherent	1–2–3–7–8–9–10–12–13–16–17–18–19–20–21–22–23–25–27–28–29–30	38.4 ± 7.2	22

The Pearson correlation coefficient between FIQR and the A-14 Scale (−0.2827), fails to identify a linear correlation between the two.

### Assessment of knowledge about FM

At the beginning of each interview, subject knowledge of FM was first assessed in order to determine their awareness of the disease (i) etiology, (ii) main symptoms, and (iii) which treatments are considered effective. Although there are symptoms commonly reported by subjects, including pain and/or muscle stiffness, as well as difficulty concentrating, each of them had a very personal and specific experience of FM. This finding highlighted even more the need to personalize each interview based on the issues that emerged.

In general, knowledge about fibromyalgia was rather poor. In fact, when asked to explain what they knew about the disease, the vast majority of subjects (*n* = 27) did not know which words to use to describe it; consequently, these subjects were provided with information about FM and factors involved in the modulation of pain. Interestingly, almost all subjects (*n* = 28) linked the appearance of symptoms to specific traumas suffered in their life, such as surgery (*n* = 1), difficult childbirths (*n* = 1), loss of a loved one (*n* = 25), or stressful life moments (*n* = 1). In all cases, symptoms appeared two to three months after the trauma. Only one person (*n* = 1) was unable to identify any particular moment attributable to the onset of symptoms.

The next part of the educational intervention focused on the three fundamental pillars of FM management (Sarzi-Puttini et al. 2020) which include: (i) education, (ii) non-pharmacological treatments and (iii) pharmacological treatments. In this part of the interview, it emerged that all subjects had already tried many non-pharmacological treatments, the more effective ones being (i) yoga and pilates (*n* = 22), (ii) cognitive-behavioural therapies or psychological paths for the acceptance of the condition (*n* = 30) and (iii) exercising in hot water (*n* = 3). Most people were physically active (*n* = 28). Only two subjects were physically inactive despite a declared awareness of the benefits of physical activity for symptom control.

### Assessment of knowledge about MC

At the time of the interview about one third of the patients (*n* = 8) were taking MC-based drugs. Two other patients had previously taken it but one stopped the therapy due to intolerance of side effects, and the other because of the exorbitant cost of the preparation. The majority of subjects (*n* = 20) had never taken MC-based preparations but were interested in this type of therapy and in receiving more information.

All patients currently on MC therapy said that MC helped to improve their quality of life. They reported that the self-management of drug posology resulted in an optimal management of symptoms.

People who had never taken cannabis preparations before (*n* = 20) were counseled on what MC and cannabinoids are, and why they were used in the treatment of FM. In particular, the nature of synthetic cannabinoids and their preparations was explained in the context of phytocannabinoid extracts.

### Problems that emerged caused by FM

All subjects identified that their diagnosis of FM occurred several years after the first clinical visits. Moreover, all subjects stated that their physicians appeared to have little knowledge about FM which consequently undermined their trust in physicians to which they were subsequently referred and presumed a lack of physician support. This corrupted trust was reinforced in all subjects by previous diagnoses of insanity, psychosomatic illness, intolerance of discomfort and underestimates of their condition’s severity. The deficient empathy of their condition, and marginal support, by immediate family members (e.g. spouses, *n* = 20) was regarded as especially debilitating. It is not surprising that all subjects sought psychological and/or psychiatric treatment. Furthermore, almost all subjects (*n* = 28) identified ineffective initial briefings about FM during their initial clinical visits such that although the concept of “*tender points*” was familiar, subjects remained naive to the origin of their pain. Consequently, some subjects (*n* = 5) began antidepressant treatment while oblivious to its role in pain modulation.

It was especially problematic that the National Health System failed to officially recognize FM as a genuine disease.

Finally, all subjects declared employment difficulties, including multiple successive jobs, arising from unduly difficult physical requirements which were compounded by difficulties in accessing and financing medical diagnostic appointments.

### Problems that emerged caused by MC

There are significant difficulties associated with recruitment, prescription and information related to MC.

Subjects not on MC therapy (*n* = 20) declared that, despite the many years of ineffective standard therapies, their General Practitioner (GP) never offered them cannabis as a supportive therapy. By contrast, subjects receiving MC therapy (*n* = 8) identified that it was their GP who proposed it, or that the subject requested it from their GP, following suggestions from friends or acquaintances who were already using it. All subjects stated that exorbitant drug costs constitute a major restriction to MC therapy as it is not reimbursed by the National Health System; to illustrate, one subject opted to reduce their MC dosage for economic reasons. Physician resistance to MC therapy remains a major stumbling block to FM patient management; for example, of the subjects enrolled in MC therapy (*n* = 8), two identified prolonged resistance on the part of their GP to begin their MC therapy (these GPs had prejudices and hesitations in prescribing and little knowledge of the scientific evidence and of legislation). Additionally, many subjects (*n* = 12) reported GP reluctance and cited that MC therapy was the responsibility of the referred rheumatologist. Restrictions associated with the COVID-19 pandemic further limited the availability of MC as well as patient (*n* = 2) access to rheumatologists to source a prescription for MC.

### Post-intervention clinimetric data

Results of the post-intervention FIQR are summarized in Table 4.

**Table 4:** Means, standard deviations and numerosity of post-intervention FIQR and relative severity index

Severity Index	Patient code	FIQR Result post-intervention	Numerosity
Remission	16^−^−17^−^	20 ± 10.6	2
Mild	3^−^−4–5–7^−^−11^−^−19−21^−^	40.3 ± 3.2	7
Moderate	2–9^−^−15^+^−22^+^	53.2 ± 6.9	4
Severe	1^+^−6^−^−10–12–14−18^−^−20^+^−23−25^−^−26–27–28^−^−29−30	75.4 ± 4.3	14
Very Severe	8^+^−13^+^−24^+^	91.2 ± 2.9	3

- indicates a shift to a lower severity index than pre-intervention

+ indicates a shift to a higher severity index than pre-intervention

Educational intervention was followed by reduced scores in two subjects compared with an improvement in severity index in 14 subjects. The average post-interview FIQR score was 62.15 with a severity index identified as “moderate”. The Student T-test calculated for the FIQR shows a statistically significant difference compared with pre-test results (*p* < 0.001).

A comparison of data in Table 5 with those in Table 6 illustrates how the total number of adherents to MC therapy (*n* = 8) remained unchanged, but the average score improved to 44.03.

**Table 5:** Means, standard deviations and numerosity of A-14 scale questionnaire post-educational interview

Adherence	Patient code	A-14 Scale Result	Numerosity
Adherent	2^§^−4–5–11–12–14−22^§^−24−26	53.2 ± 3.2	9
Non adherent	1–3−6^§^−7–8–9–10–13−15^§^−16–17–18–19–20–21–23–25–27–28–29−30	40.1 ± 6.2	21

§ indicates a shift of the patient to an adherent/non-adherent MC therapy vs. pre-intervention

The A-14 Scale values associated with pre- and post-intervention were statistically significantly (*p* = 0.02) different. Post-intervention FIQR and The A-14 Scale scores were weakly and negatively related to each other (Pearson correlation coefficient = −0.1129). Table 6 shows the Clinical Global Impression - Global Improvement (CGI-I) Scale, which subjectively tracks the patient’s progress, administered after the educational intervention. Responses included (i) non-assessable (*n* = 8), (ii) slightly improved (*n* = 11), (iii) improved (*n* = 4), and (iv) unchanged (*n* = 7).

**Table 6:** CGI-I scale results post-intervention

Compared to your baseline condition, how much do you feel changed?	*N*° patients
0 = Not assessable	8
1 = Slightly improved	11
2 = Improved	4
3 = Much improved	0
4 = No change	7
5 = Slight deterioration	0
6 = Worsened	0
7 = Much worse	0

## Discussion

This study investigates the influence of nurse-mediated educational intervention on FM-patient awareness of MC-therapy. Subject responsiveness to the educational intervention was assessed by the application of online clinimetric tests before and after the intervention. The online approach subverted barriers to subject participation associated with geographic distance, and societal restrictions associated with the COVID-19 emergency.

A comparison of FIQR and the A-14 scale scores before and after the educational intervention indicates a statistically significant improvement in the subject condition. Similarly, CGI-I scale results also indicate subject improvement; specifically, 10 subjects declared a slight, albeit significant, improvement following the educational intervention whereas four subjects identified a marked improvement.

Interviews revealed that all subjects were dissatisfied with their ongoing control of FM symptoms and pain despite conspicuous pharmacological intervention. All subjects identified that the 30-minute interview, with associated information, was helpful; in particular, there was universal identification of the importance for subjects to be heard and their condition and symptoms acknowledged. Subject disappointment and discouragement due to societal and medical-community ignorance and lack of recognition of their condition was evident; a situation not restricted to Italy (Briones-Vozmediano 2017).

In Italy, there is no uniform national-level direction or legislation about the use of medical cannabis and associated activities such as motor vehicle operation or employment. Medical cannabis is still perceived and recognized as a “recreational drug” incompatible with working life, and knowledge about MC among healthcare professionals is still lacking (Fitzcharles et al. 2014). These factors, as well as its exorbitant cost, heavily affect the decision to prescribe and to take MC. Moreover, the high costs make MC preparations prohibitive for many patients.

Subject interview narratives also revealed that few patients knew of the possibility of using preparations containing MC when other pharmacological approaches have failed to control symptoms.

Many respondents (*n* = 12) reported that their GPs were not willing to prescribe cannabis preparations as they believed it is the responsibility of the referring rheumatologist. This observation highlights that the complex prescription process, as it is in Italy, is in some ways hampering healthcare professionals from prescribing MC, and that there is a pressing need for sharing information between GPs and Rheumatologists to provide integrated care for FM patients.

A preeminent finding of this study is the urgent need for FM patients, as well as physicians, to be able to find credible and reliable sources of information on FM. The inadequate awareness of FM by physicians has contributed to an unduly distracted and prolonged diagnostic process resulting in FM patients who feel abandoned; consequently, these patients are predisposed to making inconsistent decisions accompanied by low adherence and compliance with therapy. Starting a psychological support path because they are considered “crazy” is one of the most limiting factors in improving patients’ well-being. This study showed that taking the time (which was not excessive but stayed within 30 min) to explain to patients what fibromyalgia consists of, how it manifests, and which pharmacological and non-pharmacological approaches can be used to mitigate symptoms, was itself very reassuring for patients, and this is a constant datum that is present in the literature, since the latest EULAR recommendations started from patient’s education for FM treatment (Macfarlane et al. 2017).

### Limitations

Limitations of the present study are the small sample size and the short duration of the follow-up (two weeks). Regarding the selection process, there may have been a selection bias, which included in the study just those patients who were not satisfied with their current treatment and therefore had a higher perceived effect of the educational intervention. However, this selection process was necessary to guarantee patients’ privacy.

## Conclusion

Our results suggest that it is very important to include patient education carried out by professional nurses with comprehensive knowledge on fibromyalgia and therapeutic options in the therapeutic pathway of FM patients, from the first access and for the entire duration of the care.

## Data Availability

The data presented in this study are available on request from the corresponding author due to ethical reason.

## Abbreviations

AISF: Italian Association of Fibromyalgia Syndrome
CGI-I: Clinical Global Impression-Global Improvement scale
COREQ: Consolidated criteria for Reporting Qualitative Research
EULAR: European League Against Rheumatism
FIQR: Revised Fibromyalgia Impact Questionnaire
FM: Fibromyalgia
GP: General Practitioner
MC: Medical cannabis
